# Silencing of *HaAce1* gene by host-delivered artificial microRNA disrupts growth and development of *Helicoverpa armigera*

**DOI:** 10.1371/journal.pone.0194150

**Published:** 2018-03-16

**Authors:** Ravi Prakash Saini, Venkat Raman, Gurusamy Dhandapani, Era Vaidya Malhotra, Rohini Sreevathsa, Polumetla Ananda Kumar, Tilak R. Sharma, Debasis Pattanayak

**Affiliations:** ICAR-National Research Centre on Plant Biotechnology, Pusa Campus, New Delhi, India; National Botanical Research Institute CSIR, INDIA

## Abstract

The polyphagous insect-pest, *Helicoverpa armigera*, is a serious threat to a number of economically important crops. Chemical application and/or cultivation of *Bt* transgenic crops are the two strategies available now for insect-pest management. However, environmental pollution and long-term sustainability are major concerns against these two options. RNAi is now considered as a promising technology to complement *Bt* to tackle insect-pests menace. In this study, we report host-delivered silencing of *HaAce1* gene, encoding the predominant isoform of *H*. *armigera* acetylcholinesterase, by an artificial microRNA, *HaAce1*-amiR1. Arabidopsis pre-miRNA164b was modified by replacing miR164b/miR164b* sequences with *HaAce1*-amiR1/*HaAce1*-amiR1* sequences. The recombinant *HaAce1*-preamiRNA1 was put under the control of CaMV 35S promoter and NOS terminator of plant binary vector pBI121, and the resultant vector cassette was used for tobacco transformation. Two transgenic tobacco lines expressing *HaAce1*-amiR1 was used for detached leaf insect feeding bioassays. Larval mortality of 25% and adult deformity of 20% were observed in transgenic treated insect group over that control tobacco treated insect group. The reduction in the steady-state level of *HaAce1* mRNA was 70–80% in the defective adults compared to control. Our results demonstrate promise for host-delivered amiRNA-mediated silencing of *HaAce1* gene for *H*. *armigera* management.

## Introduction

World population is likely to reach nine billion by 2050 [1]. Feeding such a huge population with declining cultivable land area is a herculean task for the entire agricultural community. In such a scenario, harnessing entire genetic potential of the crop with minimum ecological disturbance is a new slogan for increasing agricultural production. Among biotic stress factors, insect-pests infestations severely limit the productivity of crops. In world, food plants are damaged by more than 10,000 species of insects, leading to yield loss as high as 60–70% [2]. *Helicoverpa armigera*, a notorious polyphagous insect, feeds on a wide variety of agriculturally important crops like chickpea, pigeonpea, sorghum, soybean, and cotton, causing serious damage to crop yield [3]. Wide host range, high mobility and fecundity, and ability to survive under adverse weather conditions are some of the reasons for the notoriety of this insect pest throughout the world. Crop losses due to *H*. *armigera* alone have been estimated to over US$2 billion annually [4].

Among several mitigation strategies, chemical insecticides are the most popular due to their rapid action and effectiveness with easy accessibility. However, their irrational application has led to numerous ecological and environmental hazards and development of resistance in targeted pests [5]. All these concerns discourage long-term use of chemical insecticides alone in future. Transgenic technology has emerged as an efficient tool for insect-pest management. Among transgenic approaches, *Bt*-technology, wherein *Bt*-genes isolated from *Bacillus thuringiensis* are genetically engineered into desired crop species, has gained tremendous success worldwide, specifically for the control of lepidopteran and coleopteran pests. However, cases of *Bt*-toxin resistance have been reported in the target insect-pest, which demand for the search of novel insects-pest control strategies to complement *Bt* technology [6].

RNA interference (RNAi), a collective manifestation of gene silencing, performs two broad functions in plant, defence against viruses, transposons and aberrant genes (endogenous or introduced transgenes) and regulation of endogenous genes involved in plant growth, development and stress responses [7–9]. RNAi has been widely used as an efficient tool in deciphering gene functions and crop improvement [10, 11]. Gene functional studies in insects have led to the discovery of essential genes which play important roles during their growth and development [12]. RNAi mediated down-regulation of such genes developed lethal phenotypes in insects, which indicated that these genes could be targeted for insects–pests control [13, 14]. Injection of si*Coo2*-RNA into pea aphids (*Acyrthosiphon pisum*) led to *Coo2* suppression and caused significant mortality of insects fed on host plant [15]. Silencing of chitin synthase gene resulted in disruption of *Spodoptera exigua* larval development [16]. Feeding of synthetic dsRNA to the diamondback moth (*Plutella xylostella*) suppressed expression of the *P450* gene resulting in reduced larval resistance to the insecticide permethrin [17]. Significant larval mortality and disruption of development of *H*. *armigera* was reported by feeding of dsRNA and artificial microRNA through artificial diet [18, 19]. In host-delivered RNAi (HD-RNAi) strategy, silencing molecules (non-coding small RNA) are produced in the transgenic host background targeting specific gene(s) of insect pest. Upon feeding on HD-RNAi plant, these silencing molecules enter the insect pest gut and silence the target gene by exploiting endogenous RNAi machinery of insect-pest. First successful demonstration of HD-RNAi mediated control of insect pest was reported in 2007 [20, 21]. Silencing of *H*. *armigera* cytochrome P450 monooxygenase gene, *CYP6AE14*, by hpRNA, delivered through transgenic tobacco, rendered the insect-pest unable to detoxify gossypol and, consequently, the insect pest showed retarded growth [20]. Significantly less root damage by western corn rootworm (*Diabrotica virgifera virgifera*) was observed in transgenic corn plants expressing dsRNA targeting the insect-pest *V-ATPase* gene [21]. Feeding of aphid (*Myzus persicae*) on transgenic tobacco and Arabidopsis expressing dsRNA targeting the aphid genes, *MpC002* and *MpRack1*, caused reduction in appearance of aphid progeny [22]. Similarly, silencing of aphid gap gene, *hunchback* (*Mphb*), by HD-RNAi mediated delivery of dsRNA caused impairment of reproductive potential of the insect-pest [23]. Feeding of whitefly (*Bemisia tabaci*) on transgenic tobacco plants expressing dsRNA against *AChE* (acetylcholinesterase gene) and *EcR* (20-hydroxyecdysone receptor gene) caused more than 90% mortality of the insect-pest [24]. Suppression of expression of three genes (*NlHT1*, hexose transporter gene; *Nlcar*, carboxypeptidase gene; and *Nltry*, trypsin-like serine protease gene) of brown plant hopper (*Nilaparvata lugens*) was achieved when BPH fed on transgenic rice plants expressing dsRNAs targeted against these genes [25]. Resistance against *H*. *armigera* had been reported by host-delivered hpRNAi-mediated silencing of *EcR*, *HR3* (hormone receptor3), *AK* (arginine kinase), *CHI* (chitinase) and *NPF* (neuropeptide F) genes [26–30].

Host delivered RNAi technology generally employs long hair-pin (hp)-RNA (dsRNA) construct to produce a pool of small interfering RNAs (siRNAs) for silencing of targeted gene(s) in the concerned insect-pest [31, 32]. Although very efficient, off-target effects and ecological safety are the major concerns against HD-hpRNA strategy as siRNA(s) might have multiple targets and may affect beneficial insects [33]**.** Artificial microRNA (amiRNA) has been used as a potent and precise gene silencing tool to circumvent the problem of off-target effects [34–37]. HD-amiRNA strategy has been successfully employed for disease resistance in plants, especially against viruses and fungal pathogens [38]. However, only a few reports are available on HD-amiRNA mediated resistance management against plant insect-pests [39, 40].

Acetylcholinesterase (AChE, EC 3.1.1.7) is an essential enzyme of the nervous system, having indispensible role in terminating nerve impulse transmission at synaptic junctions of cholinergic neurons by hydrolysing neurotransmitter acetylcholine. Its hydrolytic function is very well conserved in both vertebrates as well as invertebrates. Proper nerve impulse conduction and termination is must for coordinating various activities of an organism. In the presence of inhibitors neural circuits get deranged, and normal functions of an organism are impaired. Most of the insect species contain two different isoforms of AChE, encoded by *Ace1* and *Ace2* genes. Ace1 is identified as the dominant isoform in most of the agricultural insects-pest [41–43]. In the present study we attempted transgenic tobacco-delivered amiRNA mediated silencing of *H*. *armigera Ace1* gene, *HaAce1*. We reported growth retardation and mortality in larvae and emergence of defective adults of *H*. *armigera* due to feeding on transgenic tobacco leaves expressing amiRNA targeted against *HaAce1*.

## Materials and methods

### Plants and insects culture

Leaf discs of *in vitro* grown tobacco (*Nicotiana tabacum* L.) cv. Petit Havana were used for *Agrobacterium* mediated genetic transformation. Plants were maintained aseptically under 16 h light and 8 h dark photoperiod regime and temperature regime of 24–26°C. Well grown and hardened *HaAce1-*amiRNA transgenic and vector control (transformed with pBI121 empty vector without any *HaAce1-*amiRNA gene construct) plants were shifted to transgenic net-house under normal day-night condition for further characterization. *H*. *armigera* larvae were procured from National Bureau of Agricultural Insect Resources, Bengaluru, India **(**Accession No. NBAII-MP-NOC-01) and raised on an artificial diet and under growth condition of 26 ± 1°C, 70–80% relative humidity and 16 h light—8 h dark cycle. Larvae were reared together till second instar and separated just before the start of third instar to stop cannibalism. Male and female adults were fed on 10% sucrose solution and kept under the same growth conditions used for rearing larvae.

### Designing of artificial microRNA and construction of plant expression vector

Coding sequence of *HaAce1* (Accession no. DQ064790) was used as an input sequence for wmd2-web microRNA designer platform (http://wmd2.weigelworld.org. A freely available online tool to design amiRNAs; presently wmd3 version is available.), to get amiRNA sequences against *HaAce1*. Initially, a number of potential microRNAs targeting *HaAce1* were obtained using the amiRNA designer platform. A few amiRNAs starting with uridine residue and targeting different regions of *HaAce1* were manually selected and screened for absence of any off-targets against human, plants and beneficial insects. One of the selected amiRNAs was 5′UUAAGCUGAUCAAAAAGACCG3′ (named *HaAce1*-amiR1) targeting 633–652 nt region of *HaAce1* mRNA. *Arabidopsis thaliana* pre-miRNA164b backbone was chosen for expression of *HaAce1*-amiR1 in transgenic tobacco (S1 Fig). Recombinant *HaAce1*-preamiRNA1 was PCR amplified by extension of overlapping primers (HAR- F & R; S1 Table), ligated onto pUC19 vector and sequenced. After sequence verification, the *HaAce1*-preamiRNA1 was excised from the carrier plasmid and ligated between the CaMV 35S promoter and NOS terminator of plant binary vector, pBI121 to get the amiRNA vector cassette, pBI::HAR1.

### Tobacco transformation

*Agrobacterium tumefaciens* strain, EHA105, harbouring pBI::HAR1 was used for transformation of tobacco leaf discs. Co-cultivated explants were kept on MS selection medium containing hormones, NAA (0.1 mg/l) and BAP (2.5 mg/l) and antibiotics, cefataxime (500 mg/l) and kanamycin (100 mg/l). Selection medium was replaced after every 15–20 days to ensure proper growth and efficient selection. Adventitious shoots (nearly 5–6 cm height) were excised carefully without any callus portion and transferred to fresh selection medium for rooting after three months. *HaAce1*-amiR1 transgenic tobacco plants with well developed roots were transferred into small size pots containing soilrite for hardening, and after a few days the hardened plants were shifted to large size pots containing soil and kept in the nethouse under natural conditions.

### PCR screening and Southern analysis of transgenic tobacco

Putative transformants were screened for the presence of transgene by PCR using two different sets of primer combinations (35SP-F & NT-R1; *mirAce-*F & NT-R2; S1 Table). Genomic DNA was isolated from mature leaves following cetyltrimethylammonium bromide (CTAB) procedure [44]. Nearly 100–150 ng of genomic DNA was taken for PCR and samples were carried through 30 cycles using the following temperature cycle; 94°C for 30 sec, 55°C/60°C for 30 sec and 72°C for 1 min. Cycles were preceded by denaturation for 5 min at 94°C and followed by a final extension at 72°C for 10 min. For Southern hybridization, approximately 15 μg of genomic DNA was digested completely with high fidelity *Hin*dIII restriction enzyme (20 U/μl) (New England Biolab) at 37°C for 16–18 hours. Restricted fragments were separated on 0.8% agarose gel, blotted onto positively charged nylon membrane (Hybond N^+^, Amersham), and then exposed to UV light of 22 KJ for 90 sec. Hybridization was carried out with a PCR amplified and DIG labelled *NPTII* fragment of 700 bp. Pre-hybridization, hybridization, washings and detection were performed according to the instructions provided by DIG Luminescent Detection Kit (Roche, Germany).

### qRT-PCR and semi quantitative RT-PCR

Total RNA was isolated from *HaAce1*-amiR1 transgenic tobacco lines and *H*. *armigera* adults (deformed adults emerged after long duration feeding bioassay) using Trizol reagent (Invitrogen) as described by Molnar *et al*. [45] and treated with DNAse I using TURBO DNA-*free*™ Kit (Ambion). One microgram of total RNA isolated from tobacco transgenic line and 500 ng of total RNA isolated from *H*. *armigera* adult were reverse transcribed into cDNA, using two-step RT-PCR kit (USB). RT-PCR of total RNA, isolated from transgenic plants, was performed with *NPTII* specific primers (N- R & F; S1 Table). *Haβ*-*actin* and *HaAce1* gene specific primers (*HaAce1*- F & R and *Actin*- F & R; S1 Table) were used for qRT-PCR analysis. Reactions were performed in Mx3005P qPCR system (Stratagene) using the SYBR Green Master Mix (Takara, China) under the following conditions—initial denaturation at 95°C for 5 min, followed by 40 cycles of 95°C for 30 sec, 62°C for 30 sec and 72°C for 20 seconds. qRT-PCR of each cDNA sample and non-template control was done in triplicates. The relative expression level of *HaAce1* was calculated using 2^−ΔΔCt method and specificity of amplification was checked through melt curve analysis [46].

### Northern blotting

Small RNA fraction (size ≤ 200 nts) was separated from total RNA samples, isolated from respective transgenic and vector control leaves according to Lu *et al*. [47]. Equal amount of small RNA (nearly 36 μg) was separated on 15% polyacrylamide-urea gel and electroblotted onto the positively charged nylon membrane (N^+^, Bright Star-Plus, Ambion) by following the protocol of Rio [48]. Transferred small RNA molecules were immobilised on the membrane by EDC cross-linking as described by Paul and Hamilton [49]. Synthesized oligonucleotide (21 nt), complementary to *HaAce1*-amiR1, was labelled with DIG-dUTP using 2^nd^ generation oligonucleotide 3'- end labelling kit (Roche). Hybridization was carried out in ULTRAhyb-oligo hybridisation buffer (Ambion) containing 0.3 nanomoles of DIG end-labelled probe at 37°C for 16 hours. Membrane was subsequently washed with 2X SSC + 0.1% SDS at 28–32°C. Further steps were carried out according to the instructions provided by DIG Luminescent Detection Kit for nucleic acids (Roche, version 07).

### Insect feeding bioassays

Insecticidal activity of *HaAce1*-amiR1 tobacco transgenic lines was determined by detached leaf feeding bioassay for two different time periods: short duration bioassay for three days and long duration bioassay for approximately 20 days. Three replicates of each of the *HaAce1*-amiR1 tobacco transgenic lines, along with vector control tobacco line, were tested for insecticidal activity by short duration insect bioassay. Matured and healthy leaves (third to fifth from top) of nethouse grown tobacco lines were randomly cut and kept on moist filter paper placed in a petri plate, and five one day old second instar larvae were released into each petri plate for feeding. Filter papers were moistened at regular intervals to maintain humidity. Observations like larval growth retardation, and mortality were recorded regularly till the completion of third day. Transgenic tobacco lines showing higher level of resistance consistently in short duration bioassay were taken for long duration bioassay. Thirty synchronous second instar larvae were fed on *HaAce1*-amiR1 transgenic and vector control lines separately (one larva per plate) till their last feeding stage (sixth instar) under long duration bioassay. Fresh leaves of the respective *HaAce1*-amiR1 transgenic and vector control tobacco line were replenished throughout the period of bioassay at regular intervals. After sixth instar, larvae stopped feeding and transformed into pupae, which were collected and kept separately for adult emergence. Observations like growth retardation, mortality and developmental defects were recorded during the bioassay.

### Statistical analysis

The statistical analyses were performed using Graph Pad Prism software by one-way ANOVA. Differences between mean values of treatments were evaluated at P<0.001 significance levels.

## Results

### Design and cloning of artificial miRNA

**Fig 1:**
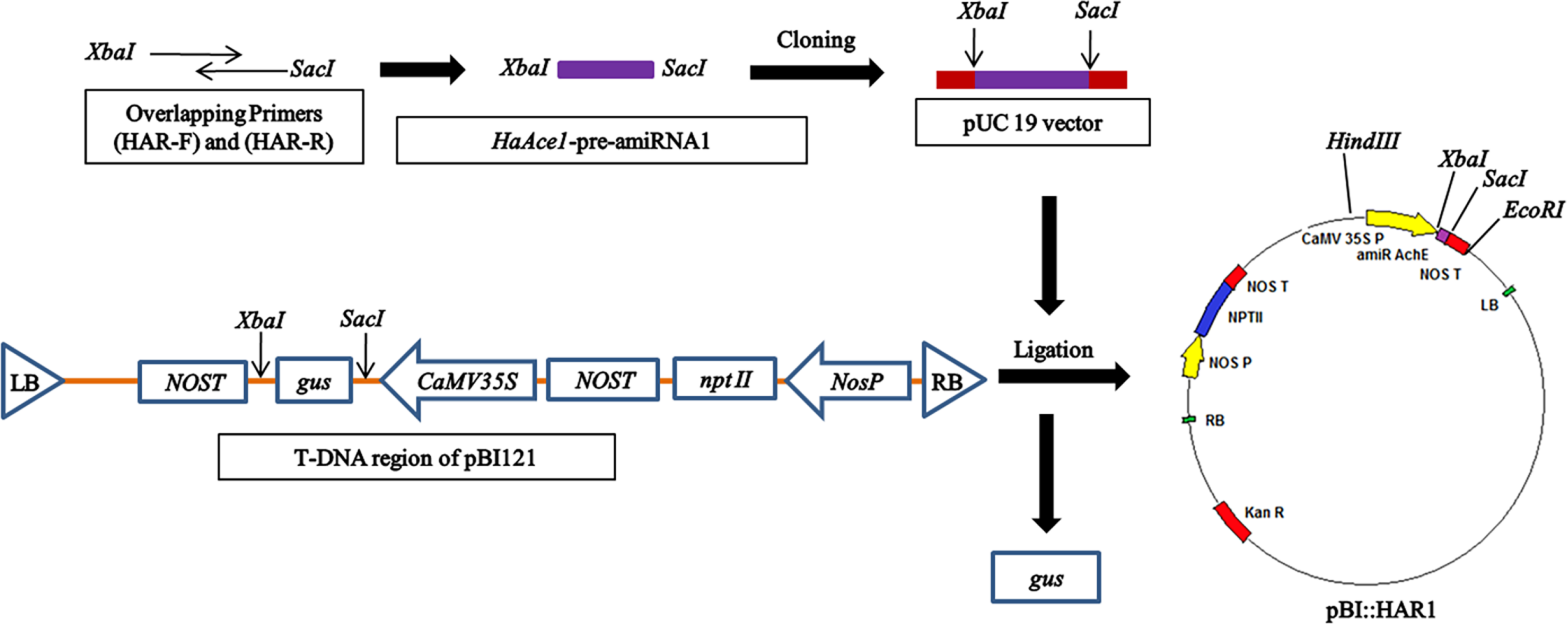
Designing and construction of pBI::HAR1. Primer extension PCR was done with a pair of overlapping primers, *HAR-*F & *HAR*-R (S1 Table) containing *Xba*I and *Sac*I recognition sequences at their 5′ ends. *Xba*I and *Sac*I digested PCR product was ligated onto pUC19. pBI121 backbone was generated by digestion with *Xba*I and *Sac*I that released *GUS* coding sequence. After sequence confirmation, *HaAce1*-preamiRNA1 DNA fragment was released from recombinant pUC19 and ligated onto pBI121 backbone to get pBI::HAR1.

The coding sequence of *H*. *armigera Ace1* gene was retrieved from NCBI and used as input for the synthesis of artificial microRNA (amiRNA) using web microRNA designer tool platform (http://wmd2.weigelworld.org). One amiRNA (5′UUAAGCUGAUCAAAAAGACCG3′; named *HaAce1*-amiR1) was chosen based on absence of any off-target against human, plants and other insects. The 21 nt long *HaAce1*-amiR1 had 20 nt complementarity with *HaAce1* mRNA spanning 633–652 nt region. The one nucleotide mismatch was at the 5′ end of *HaAce1*-amiR1 (S1B Fig). *HaAce1*-amiR1 was expressed in transgenic tobacco using the backbone of *Arabidopsis* pre-miR164b (S1C Fig). Recombinant *HaAce1*-preamiRNA1 was designed by replacing the sequence of *At*-miR164b with the sequence *HaAce1*-amiR1. The passenger strand of *HaAce1*-amiR1 (*HaAce1*-amiR1*) was designed in such a way that the original stem-loop structure remains the same (S1D Fig). The 113 bp DNA fragment encoding recombinant *HaAce1*-preamiRNA1 was synthesized through PCR, using a set of overlapping primers (HAR- F & R; S1 Table), cloned onto pUC19 vector. After confirmation of right nucleotide sequence by sequencing, the fragment was sub-cloned onto binary vector pBI121 under the transcriptional control of CaMV 35S promoter and NOS terminator to get the vector cassette, pBI::HAR1, for tobacco transformation (Fig 1).

### Generation and molecular analysis of *HaAce1*-amiR1 tobacco lines

Twenty three putative transformants were generated through *Agrobacterium-*mediated transformation of tobacco leaf-discs. Initial screening of transformants was done on MS selection medium containing kanamycin (S2A Fig). Leaf discs which appeared green after co-cultivation gave rise to callus, whereas leaf discs that turned white after co-cultivation died subsequently. The putative transformants were screened by PCR using two different set of primers (35SP-F & NT-R1; *mirAce-*F & NT-R2; S1 Table) and by RT-PCR using *NPTII* specific primers (*N*- F & R) for transgene integration and expression, respectively (S1 Table). A DNA fragment of 150 bp was amplified during PCR screening of putative tobacco transformants using mir*Ace*-F and *Nos* terminator-R1 primer combinations (S2B Fig), while an amplified product of 1 kb was obtained with *CaMV35S* promoter-F and *Nos* terminator-R2 primer combinations (S2C Fig). Out of 23 putative transformants, 22 were found positive for the presence of *HaAce1*-premiRNA1 gene sequence. RT-PCR screening with *NPTII* specific primers resulted in amplification of 700 bp DNA fragment in all the 22 PCR positive *HaAce1*-amiR1 transgenic tobacco lines (S2D Fig), and in tobacco lines transformed with pBI121 vector (vector control) (data not shown). Transgenic tobacco lines showed normal growth and development, and they are morphologically identical to untransformed control line or vector control. The 22 *HaAce1*-amiR1 transgenic tobacco lines were analysed repeatedly for insecticidal activity through detached leaf insect feeding bioassay (data not shown). During initial insect feeding bioassays two lines, 17R1 and 29R1, showed higher insecticidal activities consistently compared to that of vector control. Southern hybridization revealed that 17R1 carried single, whereas 29R1 carried two copies of the transgene (S2E Fig).

### Growth retardation and lethality in insects fed on *HaAce1*-amiR1 transgenic tobacco

Insecticidal activity was measured by analysing various parameters like growth retardation, mortality and developmental deformity. The two *HaAce1*-amiR1 transgenic tobacco lines, 17R1 and 29R1, were characterized in detail for insecticidal activity against *H*. *armigera*. Significant difference in larval growth was observed between transgenic and vector control tobacco line treated insect group during short duration bioassays. Larval growth retardation of 55% for 17R1 (P<0.001) and 35% (P<0.01) for 29R1, compared to that of control insect group was observed (S3 Fig). Larvae fed on transgenic lines showed less movement and feeding tendency over vector control fed larvae.

**Fig 2:**
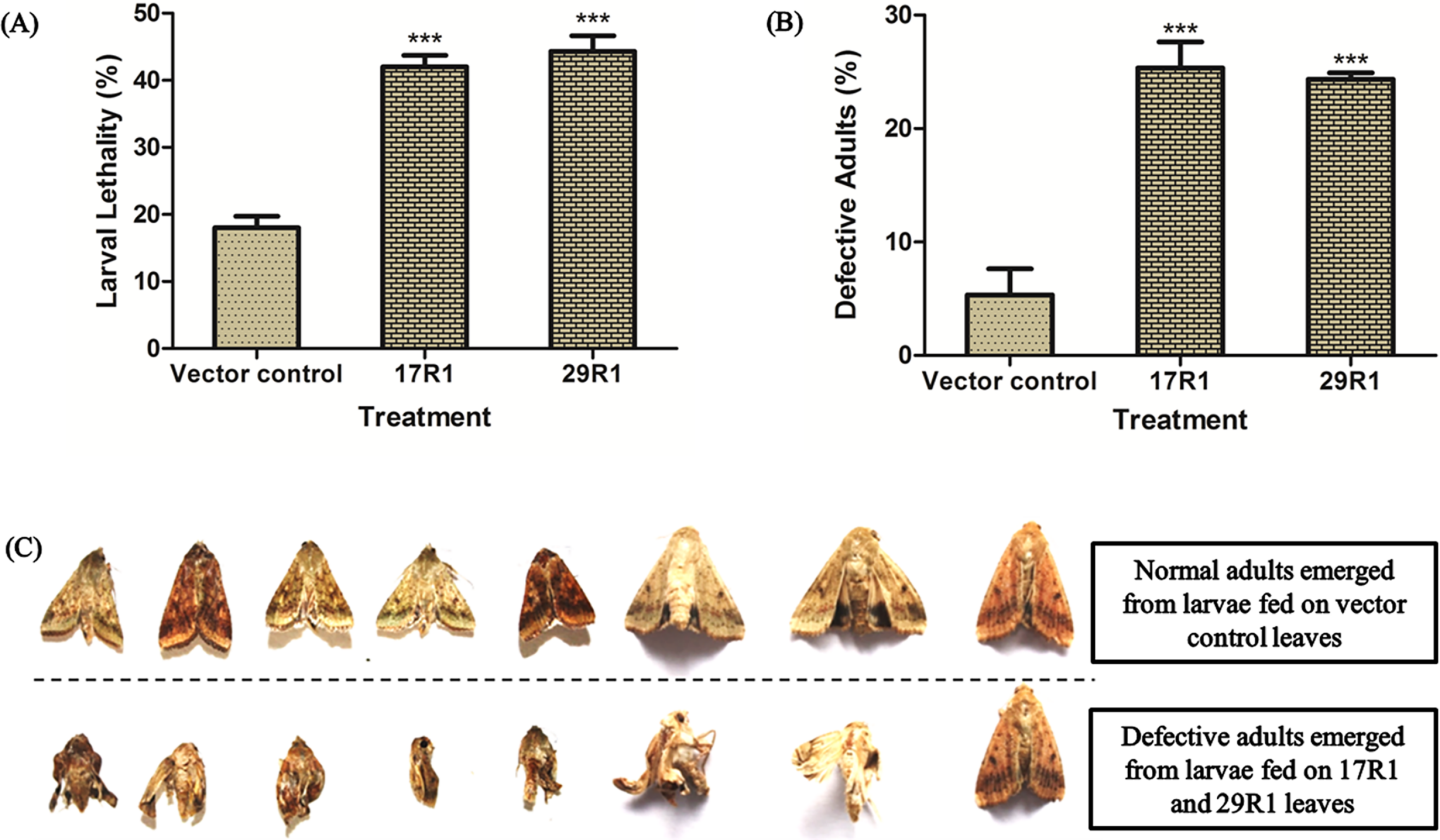
Mortality and developmental deformity in *H*. *armigera*. Thirty second instar larvae of *H*. *armigera* were fed continuously on each transgenic line (Line 17R1 and 29R1) and vector control separately (one larva per plate) till their active feeding stage**.** (A) Mortality percentage of *H*. *armigera* larvae. Vector control, mortality percentage in larvae fed on pBI121 transformed tobacco leaves; 17R1, mortality percentage in larvae fed on 17R1 transgenic tobacco line; 29R1, mortality percentage in larvae fed on 29R1 transgenic tobacco line. (B) Deformity percentage of *H*. *armigera* adults. Vector control, percentage of emergence of deformed adults from larvae fed on pBI121 transformed tobacco leaves; 17R1, percentage of emergence of deformed adults from larvae fed on 17R1 transgenic tobacco line; 29R1, percentage of emergence of deformed adults from larvae fed on 29R1 transgenic tobacco line. (C) Phenotype of *H*. *armigera* adults. Upper panel: Normal adults developed from vector control fed larvae; Lower panel: Deformed adults developed from transgenic fed larvae. One way ANOVA test was used to perform statistical analysis of the data. *** denotes extremely significant differences at P<0.001. The test was repeated three times.

Insecticidal activity of the two selected transgenic lines, in comparison with the vector control line, was further analysed through detached-leaf bioassay for long duration (20 days). Growth pattern and behaviour of *H*. *armigera* larvae were recorded during the entire course of bioassay including pupa formation and adult emergence. In general, larvae fed on 17R1 and 29R1 leaves exhibited reduced growth and less mobility compared to larvae fed on vector control tobacco leaves (S4 Fig). In some of the larvae fed on 17R1 and 29R1 tobacco transgenic leaves, appearance of bulbous, greenish to yellow jelly like substance at the anal region was observed. These larvae failed to excrete properly, stopped feeding, exhibited reduced mobility, and died later (S5 Fig). However, a few larvae, which grew normally till sixth instar stage, failed to form pupa and died (S5 Fig). Mortality rate was calculated for the entire duration of leaf feeding by larvae till the initiation of pupation. Overall mortality of 25% was observed in larvae fed on leaves of 17R1 and 29R1 transgenic lines, compared to that fed on leaves of vector control (P<0.001; Fig 2A). Larvae, which could survive, metamorphosed into pupal or chrysalis stage. Adults emerged from pupae were examined for growth and development pattern. It was observed that 20% adults, emerged from larvae fed on 17R1 and 29R1 transgenic leaves, compared to that fed on leaves of vector control, exhibited developmental deformities (Fig 2B). These adults had abnormal wing structures that impaired their movement and flying. These adult insects also had smaller body size and lower weight (Fig 2C). Differences between transgenic lines and vector control treated insect group were found extremely significant (P<0.001).

### The formation of *HaAce1*-amiR1 in transgenic tobacco and suppression of *HaAce1* expression in *H*. *armigera* adults

**Fig 3:**
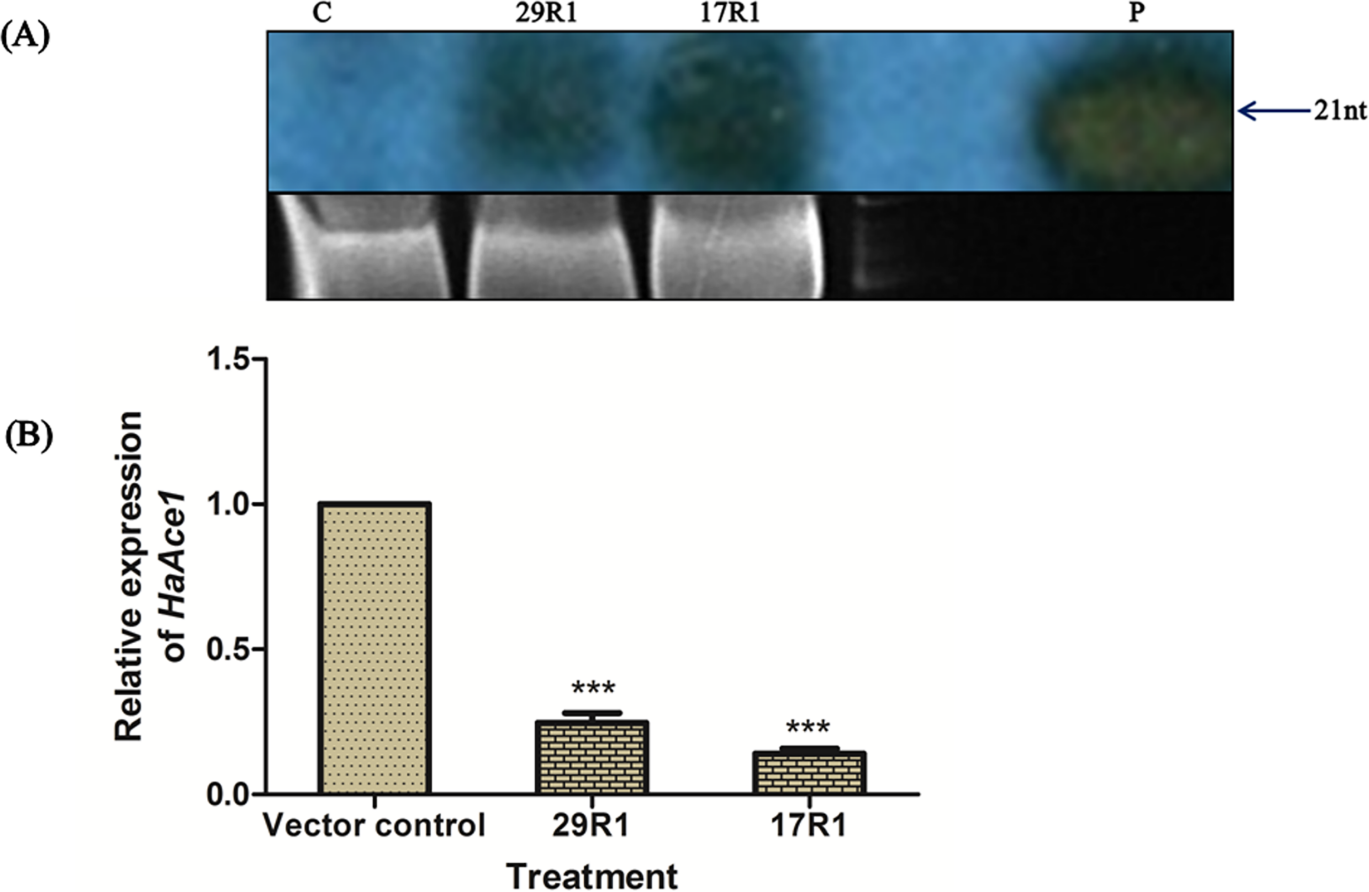
Expression of *HaAce1*-amiR1 in transgenic tobacco lines and down regulation of *HaAce1* in *H*. *armigera* adults. (A) Northern blot showing *HaAce1*-amiR1 expression in the transgenic tobacco lines, 17R1 and 29R1. Small RNAs were hybridized with DIG end labelled Anti-amiR*Ace* probe of 21 nt. C, pBI121 transformed tobacco (vector control); 29R1 & 17R1, *HaAce1*-amiR1 expression in the selected two transgenic tobacco line, 29R1 and 17R1, respectively; P, labelled Anti-amiR*Ace* probe. (B) Relative expression of *HaAce1* in the *H*. *armigera* adults. Vector control, *HaAce1* transcript abundance in *H*. *armigera* adults emerged from vector control tobacco treated group; 17R1 and 29R1, *HaAce1* transcript abundance in deformed *H*. *armigera* adults emerged from 17R1 and 29R1 transgenic tobacco treatment groups, respectively. *β*-*Actin* gene of *H*. *armigera* was used as an internal control. Real time PCR data was analysed using delta-delta Ct method. One way ANOVA test was used to perform the statistical analysis of the data. *** Extremely significant at P<0.001. The test was performed three times.

Northern hybridization was performed to detect *HaAce1*-amiR1 expression in 17R1 and 29R1 transgenic tobacco lines using reverse complement of the *HaAce1*-amiR1, Anti-amiR1, as probe (S1 Table). Anti-amiR1 was designed to have complete complementarity with *HaAce1*-amiR1. The probe was labelled with DIG-dUTP at its 3' end and hybridized with small RNA fractions of 17R1, 29R1 and vector control. A single band of 21 nt was detected in both the 17R1 and 29R1, whereas no signal was detected in vector control line (Fig 3A).

Steady state *HaAce1* mRNA levels in deformed *H*. *armigera* adults, emerged from larvae fed on 17R1, 29R1, and normal adults emerged from vector control tobacco treated larvae were analyzed through real-time PCR. *HaAce1* expression was decreased by 70–80% in deformed adult insects compared with that of adults (P<0.001) emerged from vector control treatment group (Fig 3B).

## Discussion

Studies in the past a few years clearly demonstrated that HD-RNAi technology has the potential to generate insect tolerant crops and to complement the existing *Bt*-technology in controlling agricultural insect-pests population in a sustainable way [50, 51]. However, success of RNAi technology in crop pest management depends on several factors. Selection of a gene of the target pest for silencing and a delivery method for introducing silencing molecules into the target pest are the two most important determinants of the efficiency of HD-RNAi [52]. Sensitivity of various genes to RNAi differs significantly in an organism [53]. Thus, selection of appropriate target pest gene is crucial for efficient crop protection. Generally, genes which express consistently in the relevant life stages of insect pests, and have essential functions are considered ideal for HD-RNAi silencing [21, 26, 27, 29, 54].

AChE is a highly efficient enzyme with a turnover number (Kcat) of 10^4^ substrate molecules/second, approaching the rate of a diffusion-controlled reaction [55]. This high turnover of AChE is required for precise nerve impulse termination along the neural circuits in a proper time frame. A slight reduction in the activity of the enzyme can disturb nerve impulse transmission and significantly affect the survival of an organism. Relative concentration of AChE is found to be higher in insects compared to most of the invertebrates and much higher than the vertebrates, which clearly indicates that it plays crucial role in insects [56]. Besides canonical cholinergic function, AChE also plays important roles during growth and metamorphosis of insects. Studies on RNAi mediated silencing of AChE gene demonstrated significant mortality of the targeted insect-pests. More than 90% mortality of white fly (*B*. *tabaci*) was reported after three days of feeding of transgenic tobacco leaves expressing AChE hpRNA [24]. Silencing of red flour beetle (*Tribolium castaneum*) AChE led to 100% larval mortality [43]. Aphid resistance was demonstrated in transgenic tobacco by amiRNA-mediated silencing of genes encoding AChE [39]. Down-regulation of AChE in *H*. *armigera* by employing artificial-diet based RNAi clearly demonstrated its roles in larval growth and development, pupa formation, and fecundity [18]. Between the two AChE genes, *Ace1* and *Ace2*, relative expression of *Ace1* was found to be higher in most of the lepidopteran pests, and the gene product was associated with cases of insecticides resistance [41, 57, 58]. Previously, we observed higher transcript level of *HaAce1* than *HaAce2* in *H*. *armigera* [59]. Hence, we targeted *HaAce1* for silencing considering its importance in growth and development of *H*. *armigera*.

HD-RNAi technology generally employs long hairpin (hp)-RNA construct to produce a pool of small interfering RNAs (siRNAs) for silencing of targeted gene(s) of the concerned insect-pest [31, 32]. Although very efficient, off-target effects and ecological safety are the major concerns against, HD-hpRNAi strategy as siRNA(s) might have multiple targets and may affect beneficial insects [33]. Low temperature susceptibility of plant siRNA generating mechanism is an added concern on the efficacy of HD-hpRNAi strategy all throughout the seasons in a year [60]. In contrast, amiRNA is very precise in targeting a gene that minimizes unintended silencing of off-targets, and plant machineries involved in miRNA generation appears to be temperature independent [34–37, 60]. Artificial microRNA based gene silencing was used in studying gene functionality [37, 61] and protecting plants against biotic stresses [36, 39, 62]. In fact, amiRNA was reported to be more efficient than hpRNA in silencing AChE2 gene of aphid (*M*. *persicae*) [39]. So, HD-amiRNA strategy was employed for silencing of *HaAce1* in the present study.

We selected *HaAce1*-amiR1 among the potential amiRNAs obtained employing amiRNA selection criterion (http://wmd2.weigelworld.org) due to absence of off targets in human, plants and other insect species. Initially, we aligned the amiRNAs with *HaAce1* mRNA sequence and selected a few amiRNAs with uridine at the 5′ end targeting different regions of *HaAce1* mRNA and having one nucleotide mismatch with the target mRNA at either 5′ or 3′ end of the amiRNA. The rationales behind our manual selection of amiRNAs were that majority of natural plant miRNAs have uridine at the 5′ end, and lack of perfect complimentarity between amiRNA and target mRNA minimizes RNAi transitivity [37]. Finally, we filtered the selected amiRNAs for absence of off targets by doing megablast and discontinuous megablast search of NCBI nucleotide databases. We did not find any transcript of human or plant or any other insects as potential target for *HaAce1*-amiR1. In fact, we did not observe any complementarity beyond five nucleotides between *HaAce2* and *HaAce1*-amiR1 when *HaAce2* transcript sequence was aligned with both *HaAce1*-amiR1 and its reverse complement sequences (data not shown). We chose pre-miRNA164b backbone of Arabidopsis for expression of *HaAce1*–amiR1 in transgenic tobacco as Alvarez *et al*. [35] previously demonstrated that recombinant pre-miR164b was processed precisely and efficiently in tobacco for expression of synthetic miRNA [S1 Fig]. We detected *HaAce1*-amiRNA1 of desired size (21 nt) in both the transgenic tobacco lines through small RNA northern blot analysis indicating expression and processing of recombinant pre-miR164b by tobacco endogenous miRNA machinery [Fig 3A].

We chose two best performing tobacco transgenic lines (17R1 and 29R1) for both short and long duration detached leaf feeding bioassay so that the highest possible insecticidal activity of *HaAce1*-amiR1 against *H*. *armigera* could be ascertained. Larval growth retardation of 35 to 55%, compared to control, was observed during short duration feeding bioassay of three days [S3 Fig]. However, it has been reported that extensive bioassays of long durations are more suitable in verifying the effects of RNAi based silencing on the target insect, especially on its growth and development [27]. Hence, we performed long duration insect feeding bioassay for 20 days and observed both growth retardation and mortality in transgenic line treated larvae [Fig 2]. Significant larval mortality (25%; P<0.001), observed during long duration feeding assay, could be due to the ingestion of more molecules of *HaAce1*-amiR1, which maintained their lethal concentration in the target tissue to impair both the cholinergic and non-cholinergic functions of *H*. *armigera*. Kumar *et al*. [18] reported mortality of *H*. *armigera* larvae in dose dependant manner when AChE siRNA was fed to them through artificial diet.

We also observed impairment of differentiation and development in *HaAce1*-amiR1 transgenic treated insects during long duration bioassays. Adults emerged from the pupa of *HaAce1*-amiR1 transgenic lines fed larvae had defective wings [Fig 2C]. Developmental deformity rate in adults emerged from larvae fed on *HaAce1*-amiR1 transgenic leaves was significantly higher (20%) than control insect group (P<0.001) [Fig 2B]. *HaAce1* expression was down-regulated by 70–80% in these deformed adults [Fig 3B]. Similar observation of emergence of deformed adult from larvae fed on artificial diet coated with AChE-siRNA was reported earlier [18]. Besides its role in neurotransmission, AChE also performs important role in growth, development and differentiation in invertebrates as AChE expression was detected in early embryonic stage long before differentiation of nervous system [63]. Our findings of growth retardation and mortality of larvae, and adult malformation by amiRNA-mediated specific silencing of *HaAce1* clearly indicate both the cholinergic and non-cholinergic functions of *HaAce1* in *H*. *armigera*.

## Conclusion

*Bt* has so far been the most successful transgenic technology for insect pest management [64]. However, evolution of insect species resistant to different *Bt* insecticidal proteins raises concern for continuous success of this approach. A report of analysis of 66 studies from different countries revealed field-evolved resistance in five insect-pest species to five different *Bt* insecticidal proteins [6]. This necessitates adoption of another potent insect-pest control technology that can be integrated with *Bt* technology for sustainable management of insect-pest menace. Several reports of RNAi-mediated controlling of insect-pests led researchers explore the combinatorial approach of *Bt*–RNAi for crop pest management. Next generation transgenic cotton, developed by pyramiding *Bt* and RNAi, substantially delayed evolution of insect-pest resistance compared to that of using *Bt* cotton alone [51]. Both the Canadian Food Inspection Agency (CFIA) and United States Environmental Protection Agency (US-EPA) approved commercial cultivation of a new maize event (MON 87411), the “SmartStax PRO”, expressing three *Bt* genes and a dsRNA gene construct, to control westerncorn rootworm (*Diabrotica virgifera*) [65, 66].

*H*. *armigera* causes severe economic losses by damaging several agriculturally important crops like chickpea, pegionpea, soybean, cotton etc. [3]. Hence, several attempts have been made to control this insect-pest by HD-RNAi targeting of different *H*. *armigera* genes [52]. Except one report of HD-amiRNA-mediated targeting of *H*. *armigera* chitin gene, other studies exploited HD-hpRNA-mdiated silencing of *H*. *armigera* genes. Although highly efficient, potential environmental risk of this approach is a major concern as siRNAs, generated from the hpRNA, might silence unintended genes of non-target insect species [67]. In this context amiRNA approach is a better option considering its specificity and potential of multiplexing in targeting different genes simultaneously [38, 62].

Our results showed that transgenic plant-delivered amiRNA-mediated silencing of *HaAce1* disrupted growth and development of notorious polyphagous insect pest *H*. *armigera*. The combined effect of larval mortality and adult deformity is indicative of promise of this strategy to complement *Bt* technology in controlling *H*. *armigera* menace.

## Supporting information

S1 Fig *HaAce1*-amiR1 targeting *HaAce1* of *H*. *armigera*. (A) Sequence of *HaAce1*-amiR1 (B) Homology between *HaAce1* cDNA sequence (GenBank Acc. No. DQ064790) and *HaAce1*-amiR1 reverse complement sequence. (C) Arabidopsis pre-miRNA164b; miR164b is indicated in red and miR164b* is indicated in black bold. (D) Recombinant *HaAce1*-preamiRNA1; *HaAce1*-amiR1 is indicated in red and *HaAce1*-amiR1* is indicated in black bold; two nucleotides in blue are modified in *HaAce1*-preamiRNA1 to remove *Sac*I recognition sequence (GAG CTC) present in Arabidopsis pre-miRNA164b.
(TIF)

S2 Fig Generation and molecular analysis of *HaAce1*-amiR1 transgenic tobacco lines. (A) Different stages of development of *HaAce1*-amiR1 transgenic tobacco: a. Pre-culturing of leaf-discs (explants) on pre-culture medium [MS plain with NAA (0.1 mg/l) and BAP (2.5 mg/l)]; b. *Agrobacterium* infected explants on selection medium [Pre-culture medium with kanamycin (100 mg/l) and cefataxime (500 mg/l)]; c. Initiation of adventitious shoot bud from callus; d. Well developed shoot (5–6 cm in height) transferred into rooting media [MS medium with kanamycin (100 mg/l) and cefataxime (500 mg/l)]; e. Plant with established roots; f. Plant with well developed roots shifted to pot for hardening. (B) PCR screening of putative tobacco transgenic lines with mir*Ace*-F and *NOS* terminator-R1 primers spanning 150 bp region. Lanes: L, 100 bp DNA ladder; 1–30, putative *HaAce1*-amiR1 tobacco transgenic lines; C, vector control tobacco; W, untransformed control tobacco; P, positive control. (C) PCR analysis of *HaAce1*-amiR1 transgenic tobacco lines using *CaMV 35S* promoter-F (35SP-F) and *Nos* terminator-R1 (NT-R1) primers spanning 1 kb region. Lanes: L, 1 kb DNA ladder; 1–30, *HaAce1*-amiR1 tobacco transgenic lines; C, untransformed control tobacco; P, positive control. **(**D**)** RT-PCR analysis of *HaAce1*-amiR1 transgenic tobacco lines using *NPTII* forward and reverse primers (N- F & R). Lanes: L, 1 kb DNA ladder; 1–30, *HaAce1*-amiR1 tobacco transgenic lines; C, untransformed control tobacco. **(**E**)** Southern analysis of selected *HaAce1*-amiR1 tobacco transgenic lines. Lanes: P, *Hin*dIII linearized pBI::HAR1; 17, 20, 23, 24, 29, 30, selected *HaAce1*-amiR1 tobacco transgenic lines; Lane: C, Untransformed control tobacco. Number 17 stands for 17R1 carrying single copy transgene, and number 29 stands for 29R1 carrying two copies of the transgene.
(TIF)

S3 Fig Growth retardation in *H*. *armigera* larvae during short duration bioassay. Fifteen second instar larvae were released on detached leaves of 17R1, 29R1 and vector control tobacco lines (five larvae per plate in three replications) for three days. (A) Left panel: Larvae fed on vector control leaves; Right panel; Larvae fed on transgenic lines, 17R1 and 29R1. (B) Larvae fed on transgenic leaves showed growth retardation of 35–55% compared to that of control insect group. One way ANOVA test was used to perform statistical analysis of the data. *** denotes extremely significant difference at P<0.001 whereas ** denotes very significant difference at P<0.01. The test was repeated three times.
(TIF)

S4 Fig Growth of *H*. *armigera* larvae fed on *HaAce1*-amiR1 transgenic tobacco leaves during long duration bioassay. (A) Larvae fed on vector control leaves. (B) and (C) Larvae fed on 17R1 and 29R1 transgenic lines, respectively. Thirty synchronous second instar larvae were fed on these two transgenic lines and vector control leaves continuously till their active feeding stage (one larva per plate). Larvae fed on transgenic lines exhibited retarded growth and were smaller than the vector control fed larvae.
(TIF)

S5 Fig Phenotype of *H*. *armigera* larvae fed on *HaAce1*-amiR1 transgenic tobacco (17R1 and 29R1) leaves during long duration bioassay. a-c. Greenish yellow jelly like appearance at the anal region of larvae; d-i. Less mobility in larvae; j. Sixth instar larva that suddenly stopped feeding and failed to pupate.
(TIF)

S1 Table List of primers and probe used in the current study. (DOCX)
