# Supplementary figures and images for: Silencing of HaAce1 gene by host-delivered artificial microRNA disrupts growth and development of Helicoverpa armigera
Source: PLoS One. 2018 Mar 16;13(3):e0194150. doi: 10.1371/journal.pone.0194150 (PMC5856398; doi:10.1371/journal.pone.0194150)

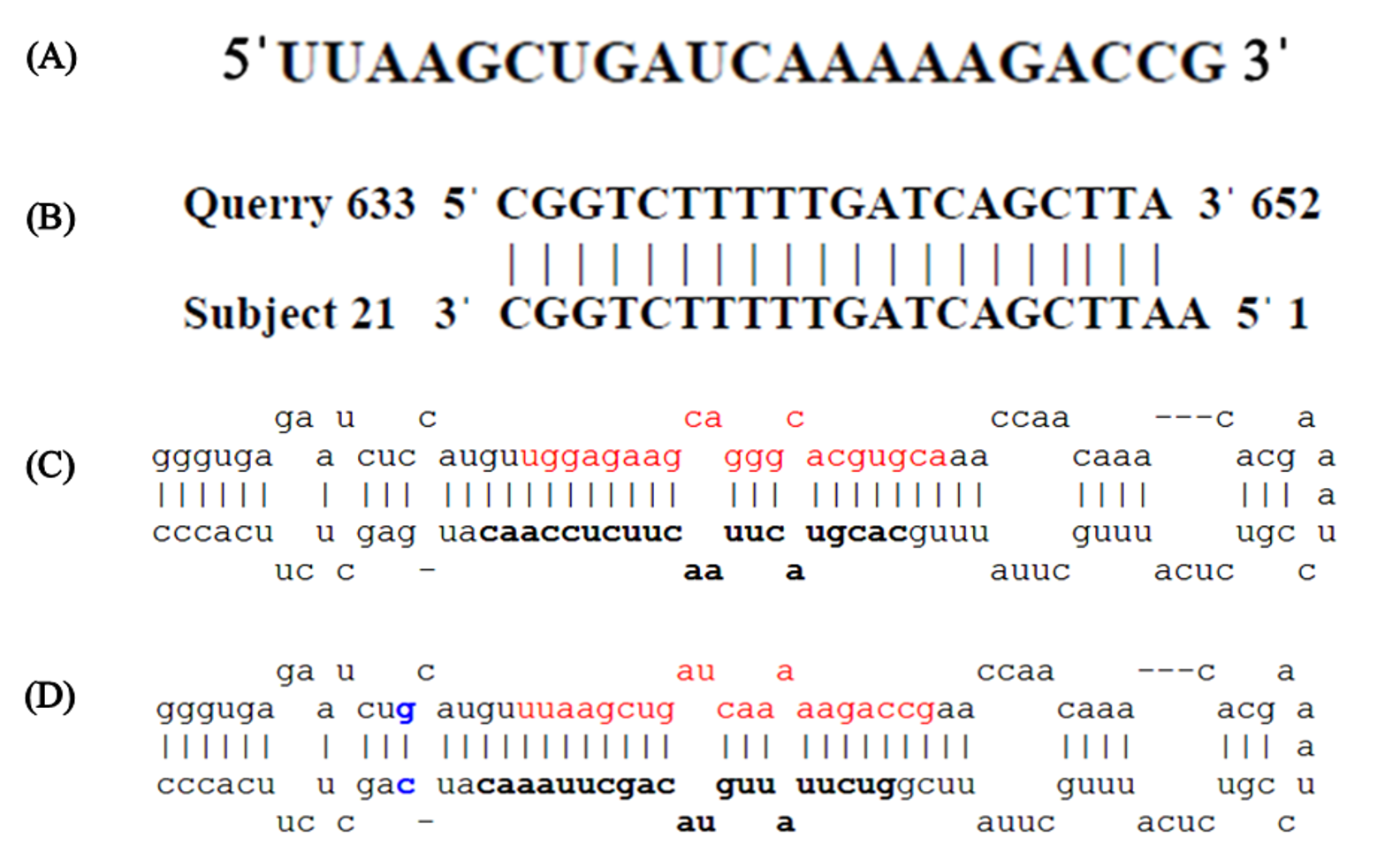

Supplement: S1 Fig — (A) Sequence of HaAce1-amiR1 (B) Homology between HaAce1 cDNA sequence (GenBank Acc. No. DQ064790) and HaAce1-amiR1 reverse complement sequence. (C) Arabidopsis pre-miRNA164b; miR164b is indicated in red and miR164b* is indicated in black bold. (D) Recombinant HaAce1-preamiRNA1; HaAce1-amiR1 is indicated in red and HaAce1-amiR1* is indicated in black bold; two nucleotides in blue are modified in HaAce1-preamiRNA1 to remove SacI recognition sequence (GAG CTC) present in Arabidopsis pre-miRNA164b. (TIF) [file pone.0194150.s001.tif]

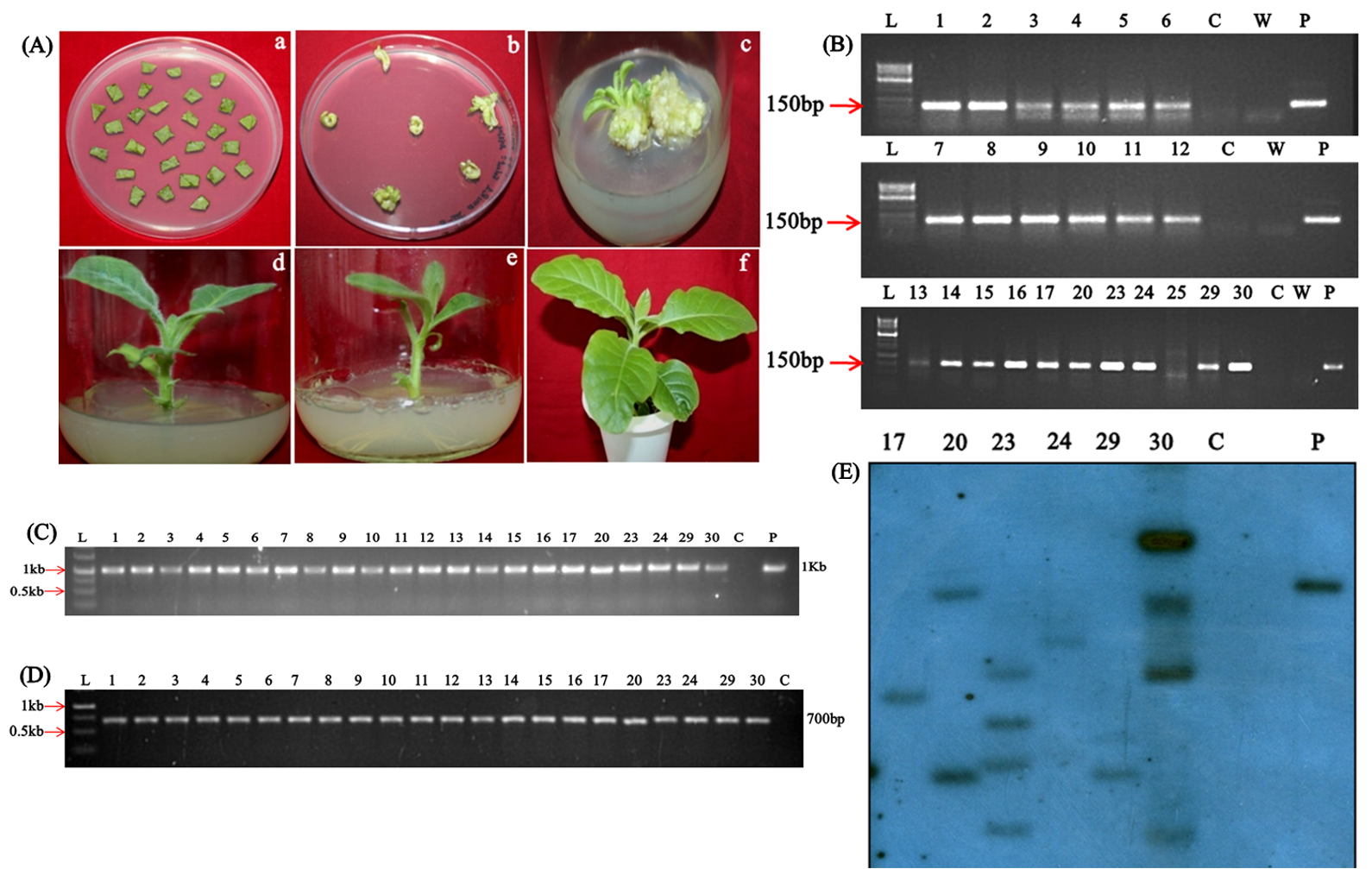

Supplement: S2 Fig — (A) Different stages of development of HaAce1-amiR1 transgenic tobacco: a. Pre-culturing of leaf-discs (explants) on pre-culture medium [MS plain with NAA (0.1 mg/l) and BAP (2.5 mg/l)]; b. Agrobacterium infected explants on selection medium [Pre-culture medium with kanamycin (100 mg/l) and cefataxime (500 mg/l)]; c. Initiation of adventitious shoot bud from callus; d. Well developed shoot (5–6 cm in height) transferred into rooting media [MS medium with kanamycin (100 mg/l) and cefataxime (500 mg/l)]; e. Plant with established roots; f. Plant with well developed roots shifted to pot for hardening. (B) PCR screening of putative tobacco transgenic lines with mirAce-F and NOS terminator-R1 primers spanning 150 bp region. Lanes: L, 100 bp DNA ladder; 1–30, putative HaAce1-amiR1 tobacco transgenic lines; C, vector control tobacco; W, untransformed control tobacco; P, positive control. (C) PCR analysis of HaAce1-amiR1 transgenic tobacco lines using CaMV 35S promoter-F (35SP-F) and Nos terminator-R1 (NT-R1) primers spanning 1 kb region. Lanes: L, 1 kb DNA ladder; 1–30, HaAce1-amiR1 tobacco transgenic lines; C, untransformed control tobacco; P, positive control. (D) RT-PCR analysis of HaAce1-amiR1 transgenic tobacco lines using NPTII forward and reverse primers (N- F & R). Lanes: L, 1 kb DNA ladder; 1–30, HaAce1-amiR1 tobacco transgenic lines; C, untransformed control tobacco. (E) Southern analysis of selected HaAce1-amiR1 tobacco transgenic lines. Lanes: P, HindIII linearized pBI::HAR1; 17, 20, 23, 24, 29, 30, selected HaAce1-amiR1 tobacco transgenic lines; Lane: C, Untransformed control tobacco. Number 17 stands for 17R1 carrying single copy transgene, and number 29 stands for 29R1 carrying two copies of the transgene. (TIF) [file pone.0194150.s002.tif]

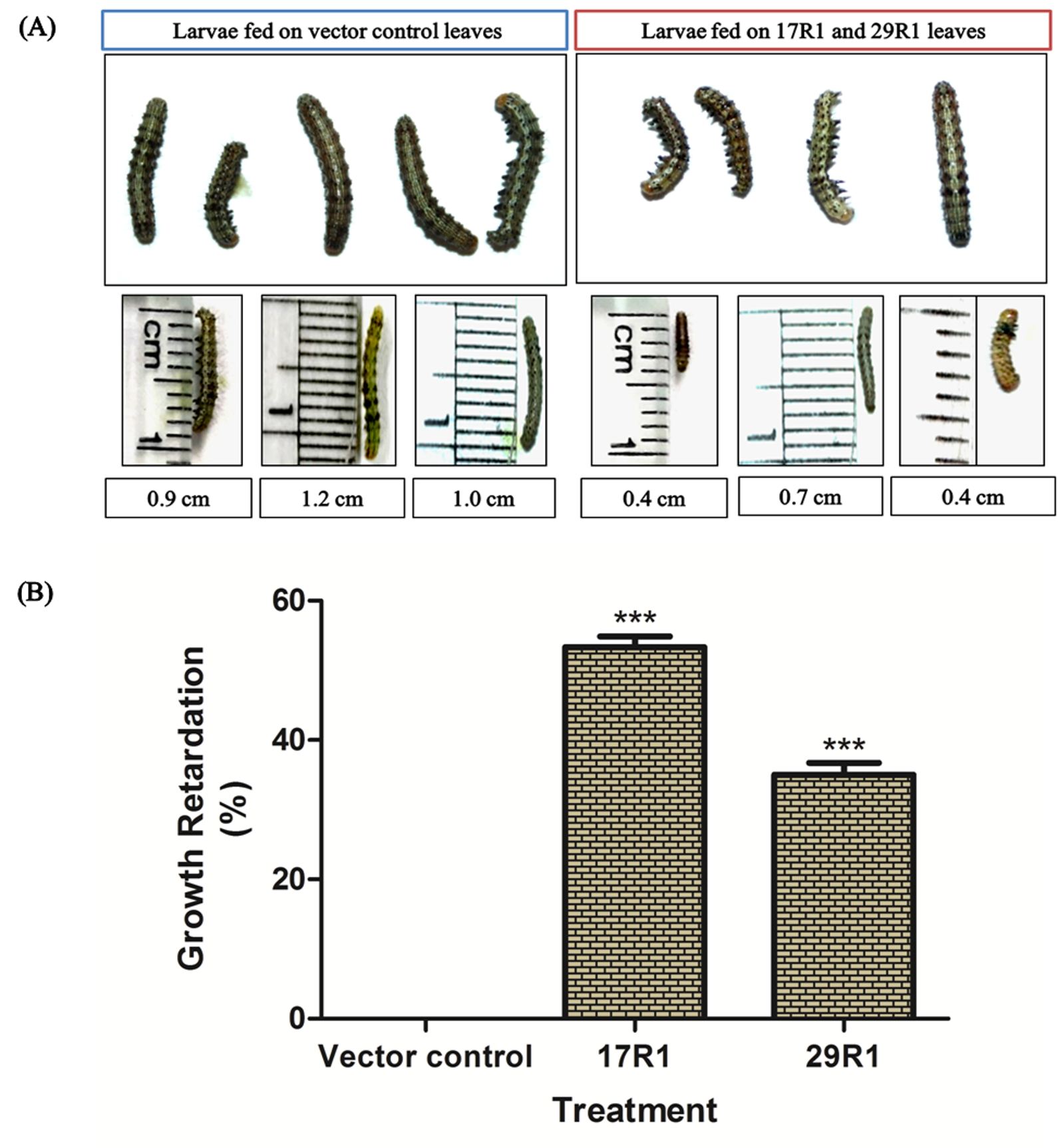

Supplement: S3 Fig — Fifteen second instar larvae were released on detached leaves of 17R1, 29R1 and vector control tobacco lines (five larvae per plate in three replications) for three days. (A) Left panel: Larvae fed on vector control leaves; Right panel; Larvae fed on transgenic lines, 17R1 and 29R1. (B) Larvae fed on transgenic leaves showed growth retardation of 35–55% compared to that of control insect group. One way ANOVA test was used to perform statistical analysis of the data. *** denotes extremely significant difference at P<0.001 whereas ** denotes very significant difference at P<0.01. The test was repeated three times. (TIF) [file pone.0194150.s003.tif]

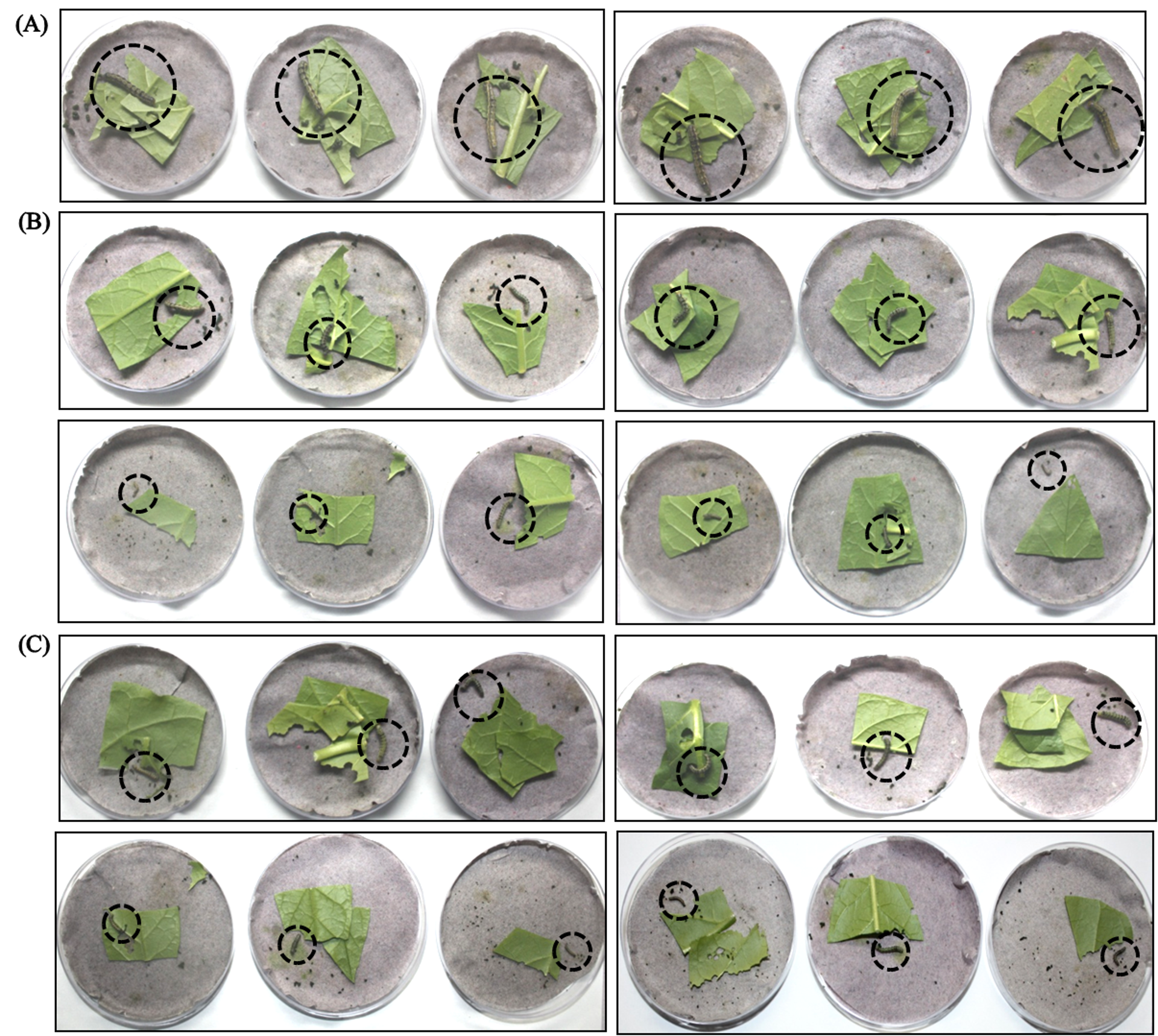

Supplement: S4 Fig — (A) Larvae fed on vector control leaves. (B) and (C) Larvae fed on 17R1 and 29R1 transgenic lines, respectively. Thirty synchronous second instar larvae were fed on these two transgenic lines and vector control leaves continuously till their active feeding stage (one larva per plate). Larvae fed on transgenic lines exhibited retarded growth and were smaller than the vector control fed larvae. (TIF) [file pone.0194150.s004.tif]

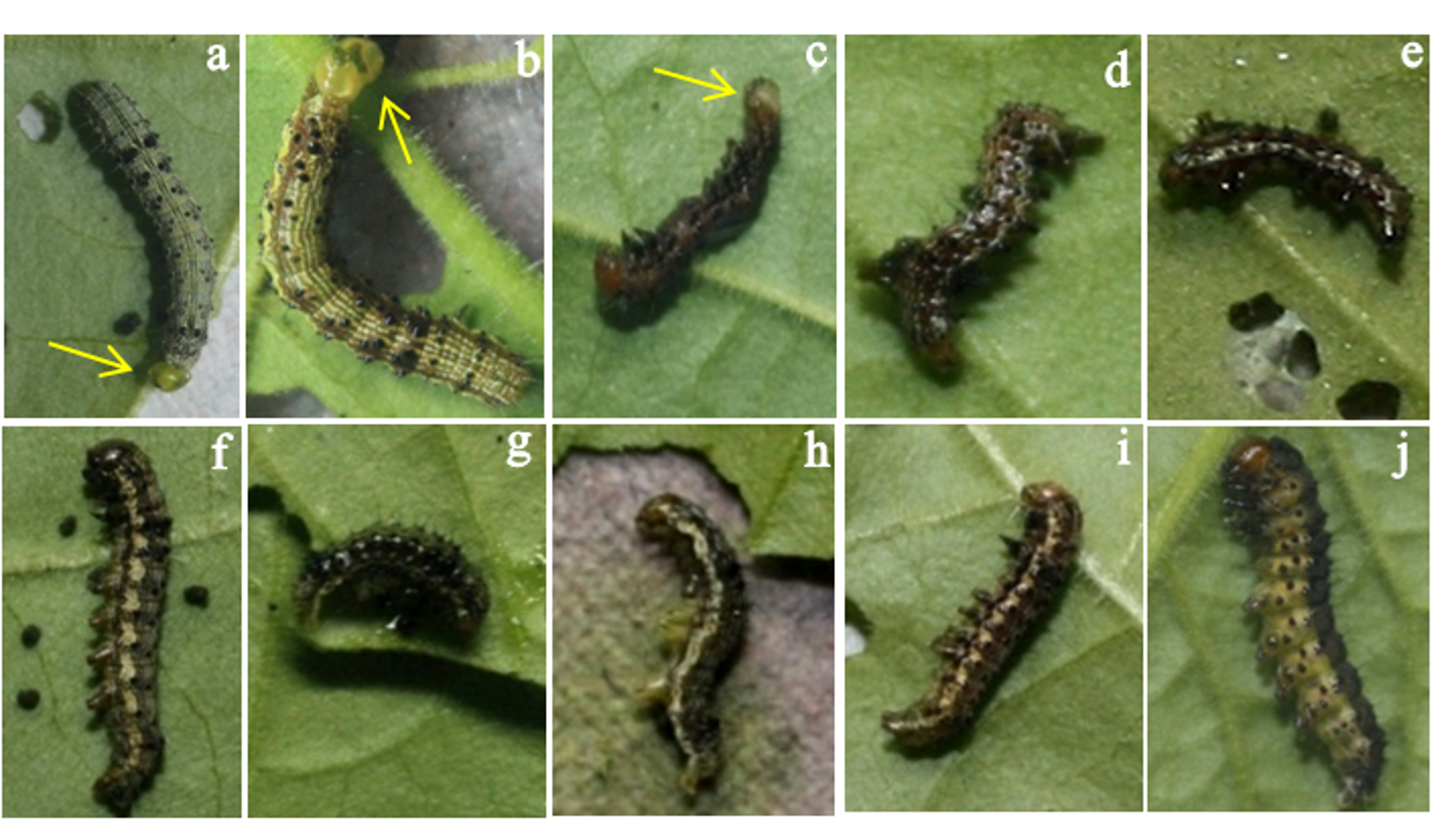

Supplement: S5 Fig — a-c. Greenish yellow jelly like appearance at the anal region of larvae; d-i. Less mobility in larvae; j. Sixth instar larva that suddenly stopped feeding and failed to pupate. (TIF) [file pone.0194150.s005.tif]
