# Supplementary material for: Silencing of HaAce1 gene by host-delivered artificial microRNA disrupts growth and development of Helicoverpa armigera
Source: PLoS One. 2018 Mar 16;13(3):e0194150. doi: 10.1371/journal.pone.0194150 (PMC5856398; doi:10.1371/journal.pone.0194150)
Supplement: S1 Table — (DOCX) [file pone.0194150.s006.docx]

**Supplementary Table 1.** **List of primers and probe used in the current study**

| Primers/Probe | Primer/ Probe Sequence |
| --- | --- |
| *HaAce1* preamiR1-F  (HAR-F)  (Overlapping sequence is indicated in red italics) | TACTAG***TCTAGA***GGGTGAGAATCTGCATGTTTAAGCTGATCAAAAAGACCGAACCA*ACAAACACGAAATCCGTCTCAT* |
| *HaAce1* preamiR1-R  (HAR-R)  (Overlapping sequence is indicated in red italics) | AAAGAC***GAGCTC***GGGTGAAGAGCTGATGTTTAAGCTGTACAATAAGACCGAATAAGCAA*ATGAGACGGATTTCGTGTTTGT* |
| *CaMV 35S* promoter-F (35SP-F) | 5′CTGCATCAAGAACACAGAGAAAG3′ |
| *Nos* terminator-R1  (NT-R1) | 5′CCTAGTTTGCGCGCTATATTT 3′ |
| *mirAce-*F | 5′AACCAACAAACACGAAATCCGTCTCATTTGCT3′ |
| *Nos* terminator-R2  (NT-R2) | 5′ATCGCAAGACCGGCAAC3′ |
| *NPTII-*F (N-F) | 5′ CCGGAATTCATGATTGAACAA3′ |
| *NPTII*-R (N-R) | 5′ CCCAAGCTTCAGAAGAACTC3′ |
| *HaAce1*-F | 5'CAGTCAACTCCAGCTCCATAG 3' |
| *HaAce1-*R | 5'AATAAGCCAGAACCTCCGAAG3' |
| *Actin*-F | 5'CCTGGTATTGCTGACCGTATGC3' |
| *Actin*-R | 5'CTGTTGGAAGGTGGAGAGGGAA3' |
| Anti-amiR*Ace* (probe) | 5'CGGTCTTTTTGATCAGCTTAA3' |
